# Supplementary material for: ZBTB12 is a molecular barrier to dedifferentiation in human pluripotent stem cells
Source: Nat Commun. 2023 Feb 9;14:632. doi: 10.1038/s41467-023-36178-9 (PMC9911396; doi:10.1038/s41467-023-36178-9)
Supplement: Supplementary file 1 — Supplementary Information [file 41467_2023_36178_MOESM1_ESM.pdf]

Supplementary Information

ZBTB12 is a molecular barrier to dedifferentiation in human pluripotent stem cells

Contents:

Supplementary Figures 1-10

Supplementary Tables 1-4

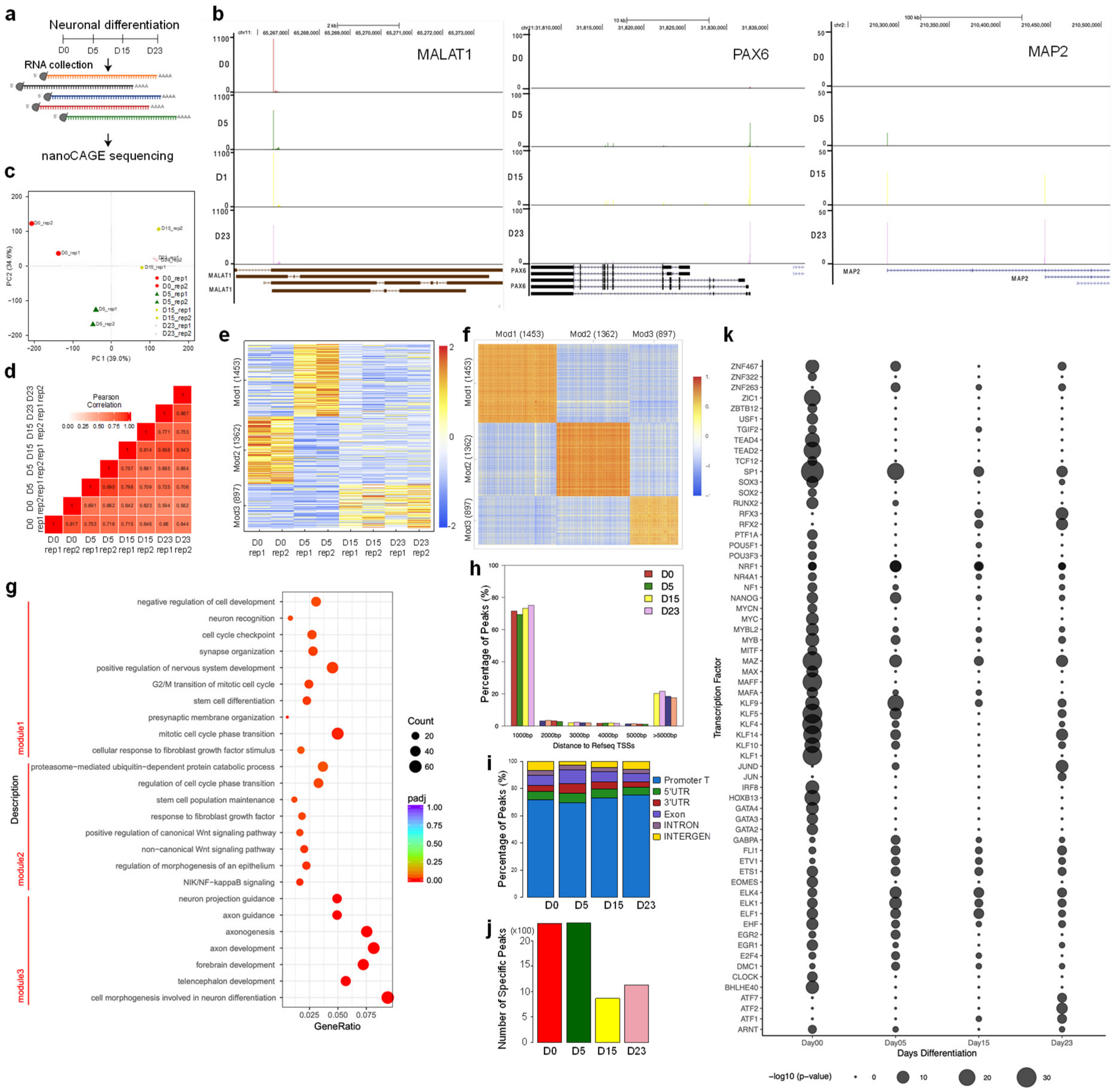

Supplementary Fig. 1 | nanoCAGE-seq analysis during neural differentiation of hESCs. **a** Schematic diagram of neuronal differentiation and nanoCAGE sequencing of H9 hESCs. **b** nanoCAGE peaks of MALAT1 (ubiquitous gene), PAX6 (neuroectoderm marker), and MAP2 (neuronal marker) during neuronal differentiation. **c** PCA plot showing clusters of 4 stage samples (D0, D5, D15 and D23) (two replicates for each stage) based on expression level measured from nanoCAGE-seq data. **d** Heatmap showing Pearson correlation of gene expression (Log_2(CPM)) through pairwise comparison for 8 samples. **e** Heatmap showing three modules obtained from 3,712 DEGs identified by EdgeR. Mod 1 (module 1) including 1,453 genes, mod 2 (module 2) including 1,362 genes, and mod 3 (module 3) including 897 genes with Q-value =0.40 (represent modularity). **f** Correlation heatmap showing three gene modules expression pattern across neuronal differential stage. **g** Bubble plot showing significantly enriched GO terms for DEGs of each module. (n= 1453 for module 1; n=1362 for module 2 and n=897 for module 3); GO term analysis was done by Hypergeometric test. *P* value of each GO term was corrected by Benjamini-Hochberg method. **h** Distance of nanoCAGE peaks from TSS (transcription start site) annotated in the human genome database (hg19). **i** Percentage distribution of nanoCAGE peaks in different genome

annotation categories. **j** Barplot showing the number of stage specific peaks from nanoCAGE data. **k** Bubble plot showing enriched transcription factors across neural differentiation stage (n=2335 for D0; n= 2345 for D5; n= 863 for D15; n= 1130 for D23), Motif was predicted compared to background sequence at each stage by HOMER software. *P* value of each predicted transcription factor (tf) was corrected by Benjamini-Hochberg method.

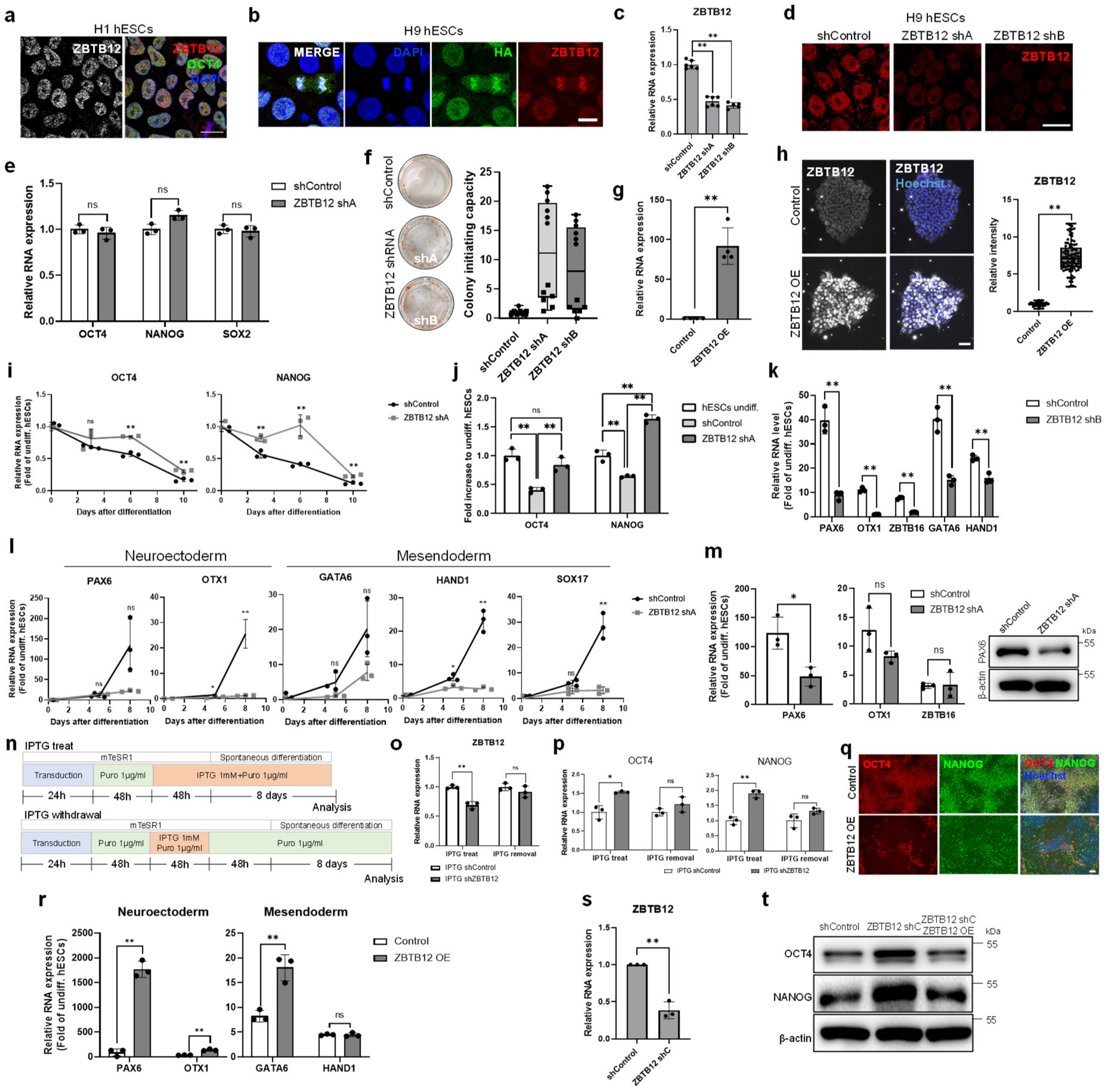

Supplementary Fig. 2 | ZBTB12 controls the balance between self-renewal and differentiation in hESCs. **a** Representative images from three independent immunofluorescence assays for ZBTB12 and OCT4 in H1 hESCs. Scale bar, 20 μm. **b** Representative images from three independent immunofluorescence assays for HA and ZBTB12 in H9 hESCs expressing HA-ZBTB12. Scale bar, 10 μm. **c, d** Knockdown efficiency of ZBTB12 shRNAs are measured by (**c**) qPCR and (**d**) immunofluorescent staining in H9 hESCs. Error bars represent mean ± sd (n=6 for shControl and ZBTB12 shA, n=5 for ZBTB12 shB). Student's *t* test (two-tailed unpaired) ** p <0.01 compared to shControl. Scale bar, 20 μm. **e** qPCR analysis of OCT4, NANOG, and SOX2 mRNA expression after ten passages of hESCs transduced with shControl or ZBTB12 shA. Student's *t*-test (Holm-Sidak's multiple unpaired). ns, non-significant. **f** Colony forming assay with H9 cells transduced with lentiviral vectors expressing either control or ZBTB12 shRNAs. Representative alkaline phosphatase (AP)-stained wells are shown. The number (colony initiating capacity) is quantified. Data are represented as box-plot with median and min to max with all data points (n=12 from two independent experiments. Independent experiments are represented by different point shapes.). Center line, median; box limits, upper and lower

quartiles; whiskers, max and min; points, all datapoints. **g** Overexpression of ZBTB12 measured by qPCR at 48 h post transduction. Error bars represent mean ± sd (n=4). Student's t test (Holm-Sidak's multiple unpaired) ** p <0.01. **h** Immunofluorescence assay for ZBTB12 in H9 cells expressing ZBTB12. N=90 from three independent experiments. Center line, median; box limits, upper and lower quartiles; whiskers, max and min; points, all datapoints. Student's t test (two-tailed unpaired) ** p <0.01. Scale bar, 50 µm. **i** qPCR analysis of OCT4 and NANOG in H9 hESCs with ZBTB12 shRNA after FGF2 and TGFβ deprivation. Error bars represent mean ± sd (n=3). Student's t test (Holm-Sidak's multiple unpaired) ** p <0.01 compared to shControl. ns, non-significant. **j** qPCR analysis of pluripotency markers (OCT4, NANOG) in differentiated (day 5) H1 cells expressing shRNAs. Error bars represent mean ± sd (n=3). Two-way ANOVA, ** p <0.01, ns, non-significant. **k** qPCR analysis of neuroectoderm (PAX6, OTX1, ZBTB16) and mesendoderm (GATA6, HAND1) markers in differentiated (day 8) H1 cells transduced with lentiviral vectors expressing shControl or ZBTB12 shB. Error bars represent mean ± sd (n=3). Student's t test (Holm-Sidak's multiple unpaired) ** p <0.01. **l** qPCR analysis of PAX6, OTX1, GATA6, HAND1, and SOX17 in H9 hESCs with ZBTB12 shRNA during embryoid body formation (Day 5 and Day 8). Error bars represent mean ± sd (n=3). Student's t test (Holm-Sidak's multiple unpaired) * p <0.05 and ** p <0.01 compared to shControl. ns, non-significant. **m** qPCR and western blot analysis of neuroectoderm markers in H9 cells expressing either shControl or ZBTB12 shA after neuroectoderm differentiation by dual smad inhibition (day5). Error bars represent mean ± sd (n=3). Student's t test (Holm-Sidak's multiple unpaired) * p<0.05. ns, non-significant. **n** Schematic diagram of IPTG-inducible ZBTB12 KD experiments. **o** qPCR analysis of ZBTB12 in H9 cells treated with IPTG or after IPGT withdrawal. Error bars represent mean ± sd (n=3). Student's t test (Holm-Sidak's multiple unpaired) ** p <0.01. ns, not significant. **p** qPCR analysis of OCT4 and NANOG in differentiated H9 cells (Day 8) expressing either IPTG-shControl or IPTG-shZBTB12. Error bars represent mean ± sd (n=3). Student's t test (Holm-Sidak's multiple unpaired) * p <0.05 and ** p <0.01. ns, not significant. **q** Representative images from three independent immunofluorescence assay for OCT4 and NANOG in differentiated (day 5) H9 cells expressing control or ZBTB12. Scale bar, 100µm. **r** qPCR analysis of PAX6, OTX1, GATA6, and HAND1 in differentiated (Day 7) H9 hESCs with ZBTB12 overexpression. Error bars represent mean ± sd (n=3). Student's t test (Holm-Sidak's multiple unpaired) ** p <0.01 compared to Control. ns, non-significant. **s** Knockdown efficiency of ZBTB12 shC that targets 3'UTR of ZBTB12 mRNA, measured by qPCR in H9 hESCs. Student's t test (Holm-Sidak's multiple unpaired). ** p <0.01 (n=3). **t** Representative images from two independent western blots for OCT4 and NANOG in differentiated (day 5) H9 cells expressing ZBTB12 shC (3'UTR targeting shRNA) with or without ZBTB12 overexpression. All data points and the exact p-values can be found in the Source Data file.

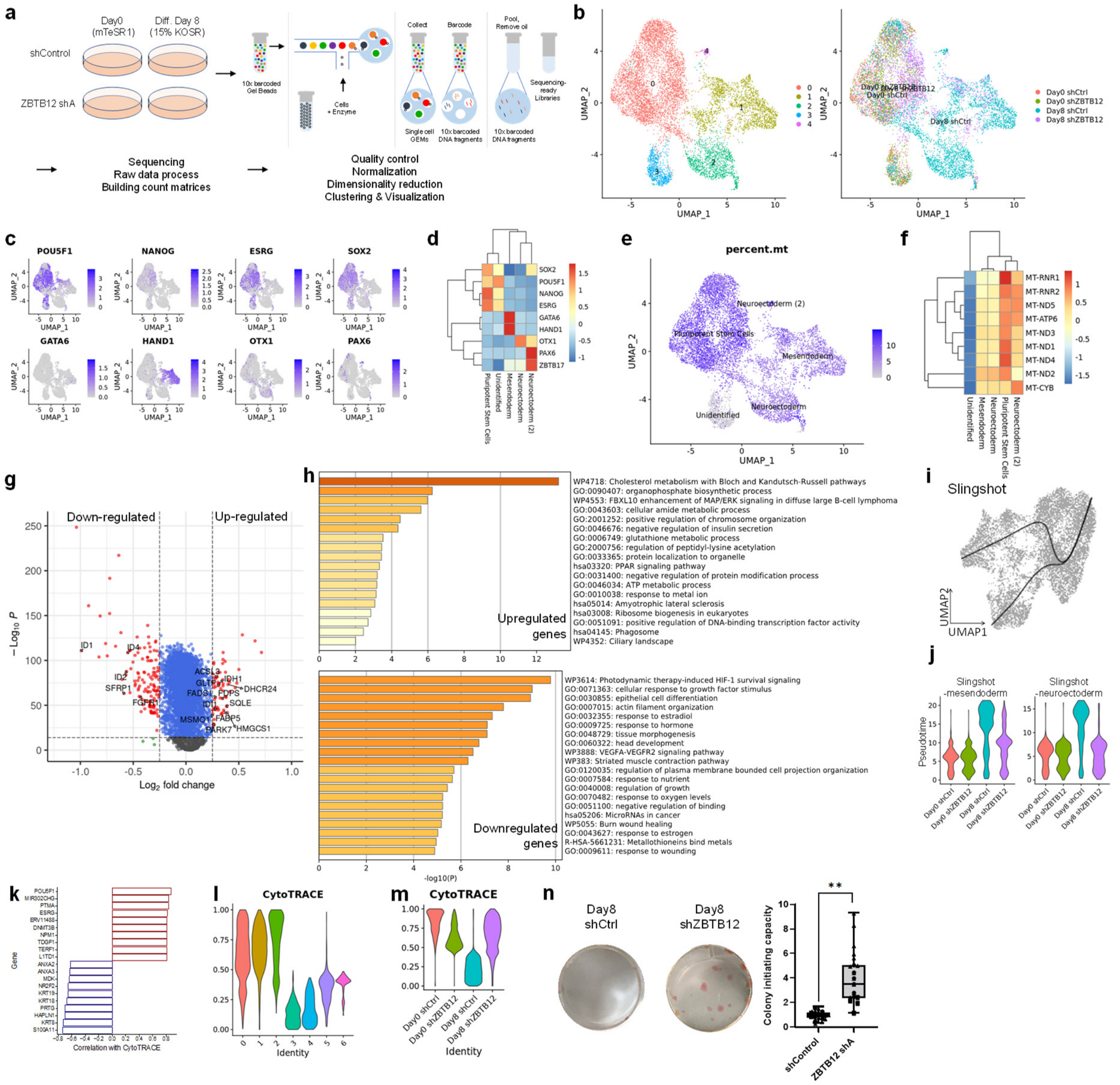

Supplementary Fig. 3 | scRNA-seq analysis confirms that ZBTB12 is essential for pluripotency exit. a Schematic diagram of scRNA-sequencing sample and library preparation, and data processing. **b** UMAP plots of 9,194 cells after filtering out cells with less than 200 genes, with UMI count higher than 30000 or with percent of mitochondrial genes higher than 15%, colored by cluster ID (left) or experimental group (right). **c, d** Marker gene expression shown by feature plot (**c**) or heatmap (**d**). **e** Cell type annotation based on marker gene expression. Cells were colored by percent of mitochondrial genes, annotating cluster 3 that shows low mitochondrial gene content as ‘Unidentified’. **f** Heatmap comparing mitochondrial gene expression level between clusters, confirming ‘Unidentified’ cluster. Cells with percent of mitochondrial genes lower than 3% are filtered out for further analysis shown in Fig. 2 and Fig. 3. **g** Volcano plot showing differentially expressed genes (DEGs, red dots) in Day0 shZBTB12 compared to Day0 shCtrl. Red dots indicate differentially expressed genes (or transcripts) with cutoff values for DEGs: $-\log_{10}P > 15$; $\log_2$ fold change < -0.25 or > 0.25. Full list of DEGs can be found in Supplementary Table 3. MAST algorithm is used to statistically determine DEGs, with Benjamini-Hochberg-corrected p values <0.05. **h** Gene ontology analysis of up or downregulated genes in Day0 shZBTB12 compared to Day0 shCtrl. Metascape calculated the statistical significance of each GO term enrichment (*p*-

value) based on the accumulative hypergeometric distribution. **i** Slingshot trajectory inference applied on UMAP. **j** Violin plots showing the distribution of pseudotime along mesendodermal or neuroectodermal trajectories within each group. **k** Genes showing the highest and lowest correlation with CytoTRACE score. Pluripotency genes such as POU5F1 (OCT4), ESRG, DNMT3B and L1TD are highly correlated to the CytoTRACE score (predicted as less- differentiated state), providing reliability of the results. **l, m** Distribution of CytoTRACE score within each cluster (**l**) or experimental group (**m**). **n** Colony forming assay of spontaneously differentiated (Day 8) H9 cells expressing either control or ZBTB12 shRNAs. Representative AP-stained wells and quantified plots are shown. Data are represented as box-plot with median and min to max with all data points (n=26, from three independent experiments represented by different point shapes). Center line, median; box limits, upper and lower quartiles; whiskers, max and min; points, all data points. Student's *t* test (two-tailed unpaired) ** $p < 0.01$. Data points and the exact *p*-value can be found in the Source Data file.

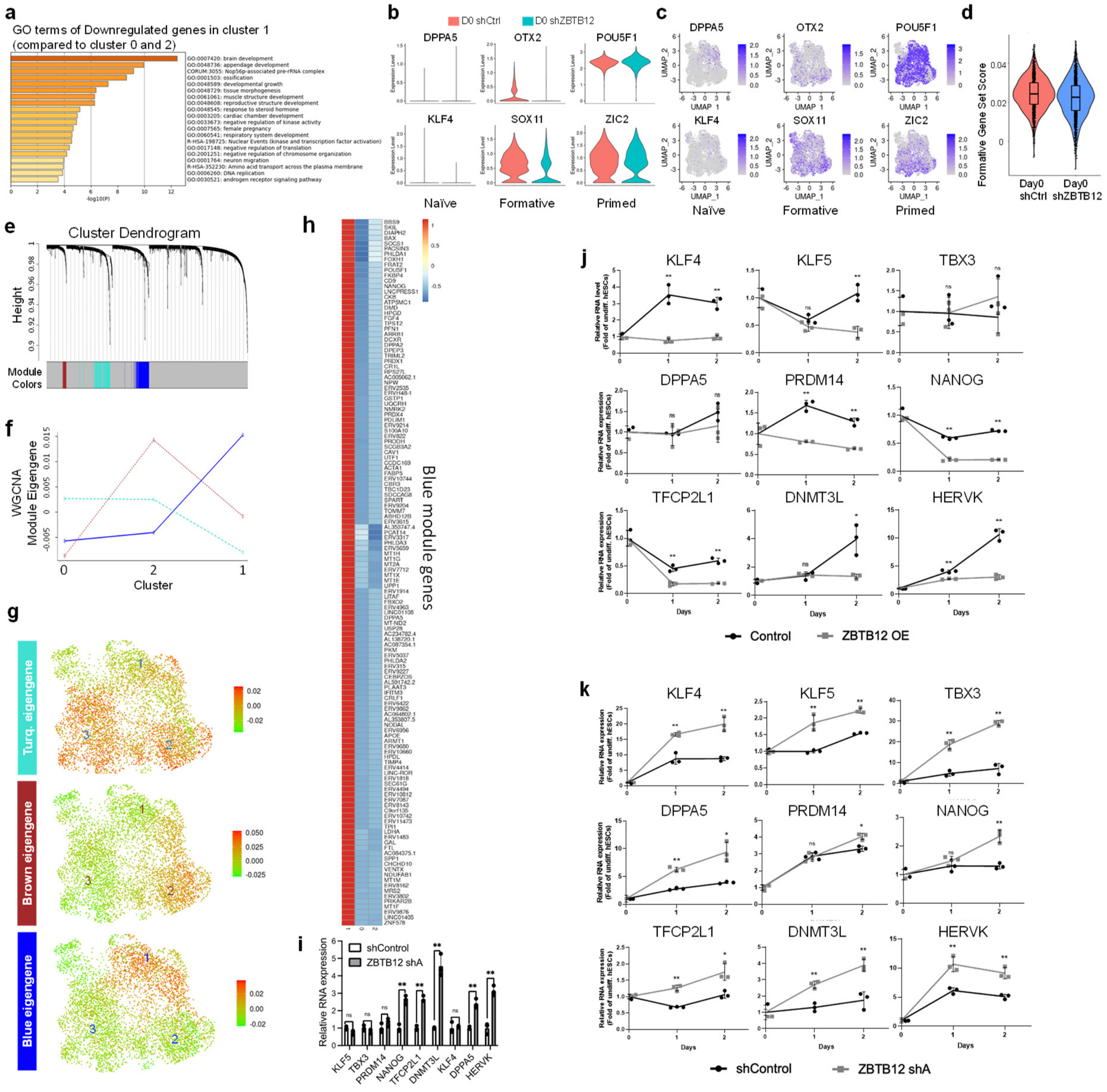

Supplementary Fig. 4 | ZBTB12 prevents de-differentiation of hESCs toward a naïve-like state. a Gene ontology analysis of downregulated genes in cluster 1 compared to clusters 0 and 2 in Fig. 3a. Metascape calculated the statistical significance of each GO term enrichment (*p*-value) based on the accumulative hypergeometric distribution. **b** Violin plots showing the expression level of naïve, formative and primed pluripotency genes in Day0 shCtrl and Day0 shZBTB12 conditions. **c** Feature plots showing the expression levels of the same genes as in (**b**) on the UMAP space. **d** Violin/box plot of formative gene set score in Day0 shCtrl and Day0 shZBTB12, determined by AddModuleScore in Seurat package. Center line, median; box limits, upper and lower quartiles; points, all data points (individual cells from one single cell experiment). **e** WGCNA cluster dendrogram identifying three (brown, turquoise, blue) gene network modules. **f** Module eigengene values of the three gene network modules in each cluster. **g** Feature plots showing eigengene values of the three gene network modules in individual cells. **h** A heatmap showing the expression level of blue module genes in three pluripotent clusters (Fig. 3a). **i** qPCR analysis of naïve marker genes in H9 cells expressing ZBTB12 shA after embryoid body formation. Error bars represent mean ± sd (n=3). Student's t test (Holm-Sidak's multiple unpaired) ** p <0.01. ns, non-significant.

j qPCR analysis of naïve marker genes in ZBTB12-overexpressing H9 cells after naive induction with LIF and 3i (PD0325901, BIO, and Dorsomorphin). Error bars represent mean ± sd (n=3). Student's *t* test (Holm-Sidak's multiple unpaired) * $p < 0.05$ and ** $p < 0.01$ compared to Control. ns, non-significant. **k** qPCR analysis of naïve marker genes in ZBTB12 KD H9 cells after naive induction by LIF and Senexin A (CDK8/19 inhibitor). Error bars represent mean ± sd (n=3). Student's *t* test (Holm-Sidak's multiple unpaired) * $p < 0.05$ and ** $p < 0.01$ compared to shControl. ns, non-significant. All data points and the exact *p*-values can be found in the Source Data file.

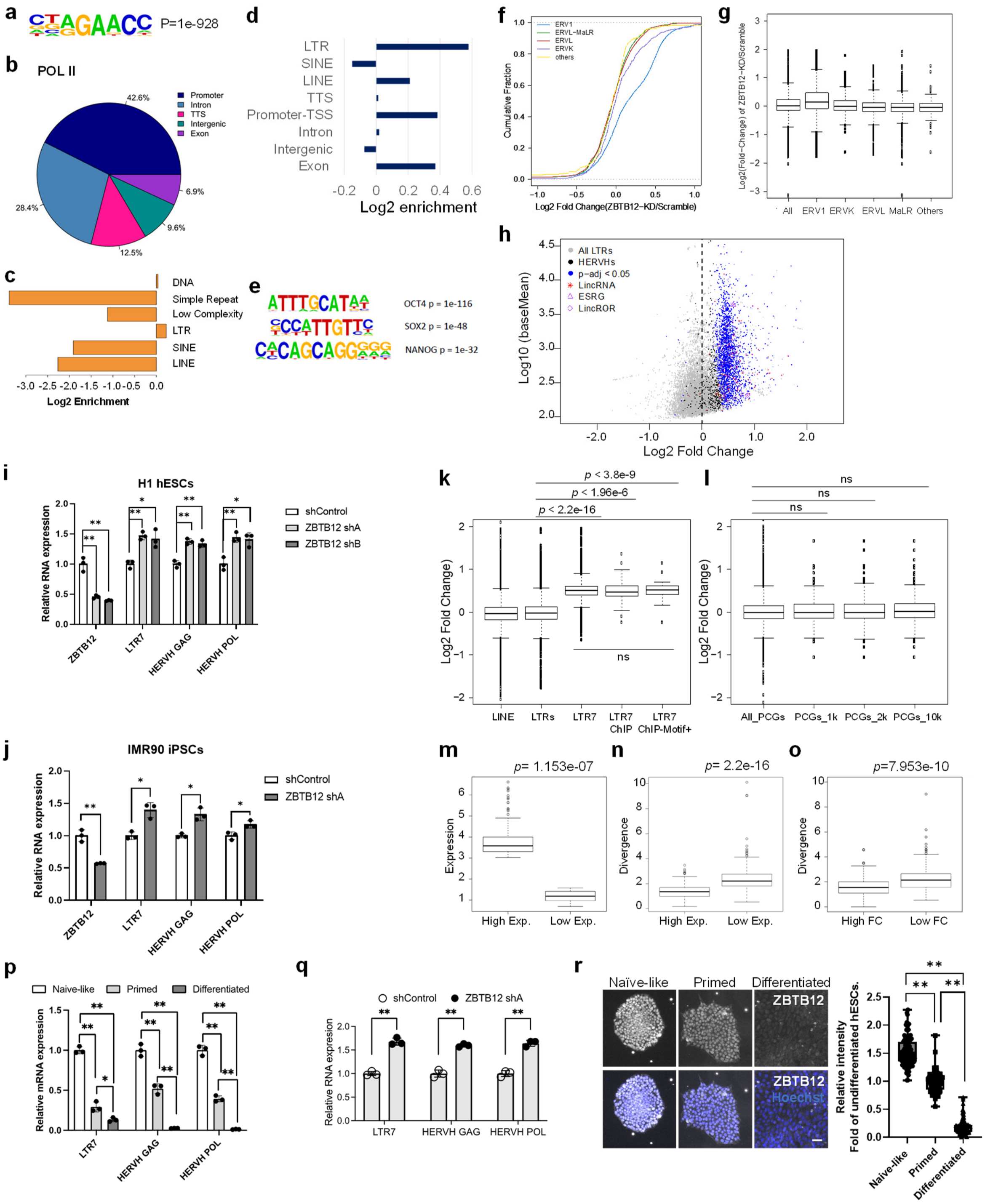

Supplementary Fig. 5 | ZBTB12 negatively regulates transcriptionally active HERVH loci. a *De novo* predicted consensus sequence from ZBTB12 ChIP-seq peaks (n=3621). Motif was predicted compared to background sequence at each stage by HOMER software. *P* value was corrected by Benjamini-Hochberg method. **b** RNA polymerase II (POL II) binding peaks distribution in different genome annotation categories. **c** Significant LTR enrichment of ZBTB12 ChIP-seq peaks among repetitive elements, when only motif-containing peaks were included. **d** LTR-enrichment of ZBTB12 ChIP-seq peaks in HEK293T (2015, Najafabadi et al.). **e** Significantly enriched NANOG, OCT4, and SOX2 binding motifs on ZBTB12 binding

sites. Motif was predicted compared to background sequence at each stage by HOMER software. *P* value of each predicted transcription factor (tf) was corrected by Benjamini-Hochberg method. **f, g** Log2 Fold Change of LTR subfamilies including ERV1 (D = 0.12954, p-value < 2.2e-16), ERVL-MaLR (D = 0.081193, p-value < 2.2e-16 for ERVL-MaLR), ERVL (D = 0.058927, p-value = 9.992e-15) , ERVK (D = 0.089826, p-value = 6.857e-08) and others (D = 0.089826, p-value = 6.857e-08) after ZBTB12 knock-down (statistical test was done by Kolmogorov-Smirnov (KS) test using each subtype compared to total LTRs, n=21265 for All; n=8223 for ERV1; n=775 for ERVK; n=3734 for ERVL; n=8280 for MaLR; n=253 for others) shown as a cumulative plot (**f**) or box plot (**g**) **h** Expression profile of individual HERV elements in H9 hESCs: Basal expression level (y-axis) and log2FC after ZBTB12 KD (x-axis). n= 18077 for All LTRs; n= 2950 for HERVHs; n=2519 for p-adj<0.05; n=123 for HERVHs overlapped with LincRNA. **i** qPCR analysis of ZBTB12, LTR7, HERVH-GAG, and HERVH-POL in H1 hESCs after ZBTB12 KD. Error bars represent mean ± sd (n=3). Student's *t* test (Holm-Sidak's multiple unpaired) * p <0.05 and ** p <0.01 compared to shControl. **j** qPCR analysis of ZBTB12, LTR7, HERVH-GAG, and HERVH-POL in IMR90 human induced pluripotent stem cells after ZBTB12 KD. Error bars represent mean ± sd (n=3). Student's *t* test (Holm-Sidak's multiple unpaired) * p <0.05 and ** p <0.01 compared to shControl. **k** Boxplot showing log2FC of indicated retrotransposons after ZBTB12 KD. LTRs, total LTR/HERVs; LTR7, total LTR7/HERVHs; LTR7_ChIP, full length HERVH loci associated with ZBTB12 ChIP-seq peaks (within 10 kb) (Two-sided two-sample KS test: D = 0.67122, p < 2.2e-16 for LTR7 vs LTRs; D = 0.64827, p < 2.2e-16for LTR7-ChIP vs LTRs; D = 0.60899, p = 3.49e-07 for LTR7-ChIP-Motif vs LTRs; D = 0.14762, p = 0.873 for LTR7-ChIP vs LTR7-ChIP-Motif; 0.14056, p = 0.8044 for LTR7 vs LTR7-ChIP-Motif; 0.10975, p-value = 0.3818 for LTR7 vs LTR7-ChIP, n=82450 for LINE; n=21267 for LTRs; n=3085 for LTR7; n=70 for LTR7 ChIP; n=21 for LTR7 ChIP-Motif). **l** Boxplot showing log2FC of protein coding genes (PCGs) after ZBTB12 KD. PCGs_1k, protein coding genes within 1kb up/downstream of ZBTB12 ChIP-seq peaks; PCGs_2k, protein coding genes within 2kb up/downstream of ZBTB12 ChIP-seq peaks (Two sided two sample KS test: D = 0.048429, p = 0.1303 for PCGs_1k vs All_PCGs; D = 0.049968, p = 0.06234 or PCGs_2k vs All_PCGs; D = 0.056889, p = 0.004842 for PCGs_10k vs All_PCGs, n=13234 for All_PCGs; n=609 for PCGs_1k; n=733 for PCGs_2k; n=1001 for PCGs_10k). ns, non-significant. **m** Boxplot showing two groups of LTR7/HERVH units (high_exp: Top 200 highly expressed units and low_exp: 200 lowly expressed units). KS test. **n** Highly expressed LTR7/HERVH units show lower divergence time compared to lowly expressed units. Divergence time of LTR7/HERVH units is calculated using Kimura 2 parameter model (human evolution rate r = 1.25*e-8). KS test, n=200. **o** LTR7/HERVH units with bigger fold-change after ZBTB12 KD show low divergence time than the ones with smaller fold-change. KS test, n=200. Center line, median; box limits, upper and lower quartiles; whiskers, 1.5x interquartile range; points, outliers for the box-plots (**g**, **k-o**). **p** qPCR analysis of LTR7, HERVH_GAG, HERVH_POL in naïve-like (3iL), primed (mTeSR1), and differentiated (KOSR, Day8) H9 cells. One way ANOVA, Error bars represent mean ± sd (n=3). * p<0.05 and ** p <0.01. **q** qPCR analysis of LTR7, HERVH-GAG, and HERVH-POL in ZBTB12 KD H9 cells after naive induction with LIF and 3i (PD0325901, BIO, and Dorsomorphin). Error bars represent mean ± sd (n=3). Student's *t*-test (Holm-Sidak's multiple unpaired) ** p <0.01 compared to shControl. **r** Immunofluorescence assay for ZBTB12 in naïve-like (3iL), primed, and differentiated (Day8) H9 cells. n=100 from three independent experiments. Center line, median; box limits, upper and lower quartiles; whiskers, max and min; points, all data points. One-way ANOVA ** p<0.01. All data points and the exact *p*-values can be found in the Source Data file.

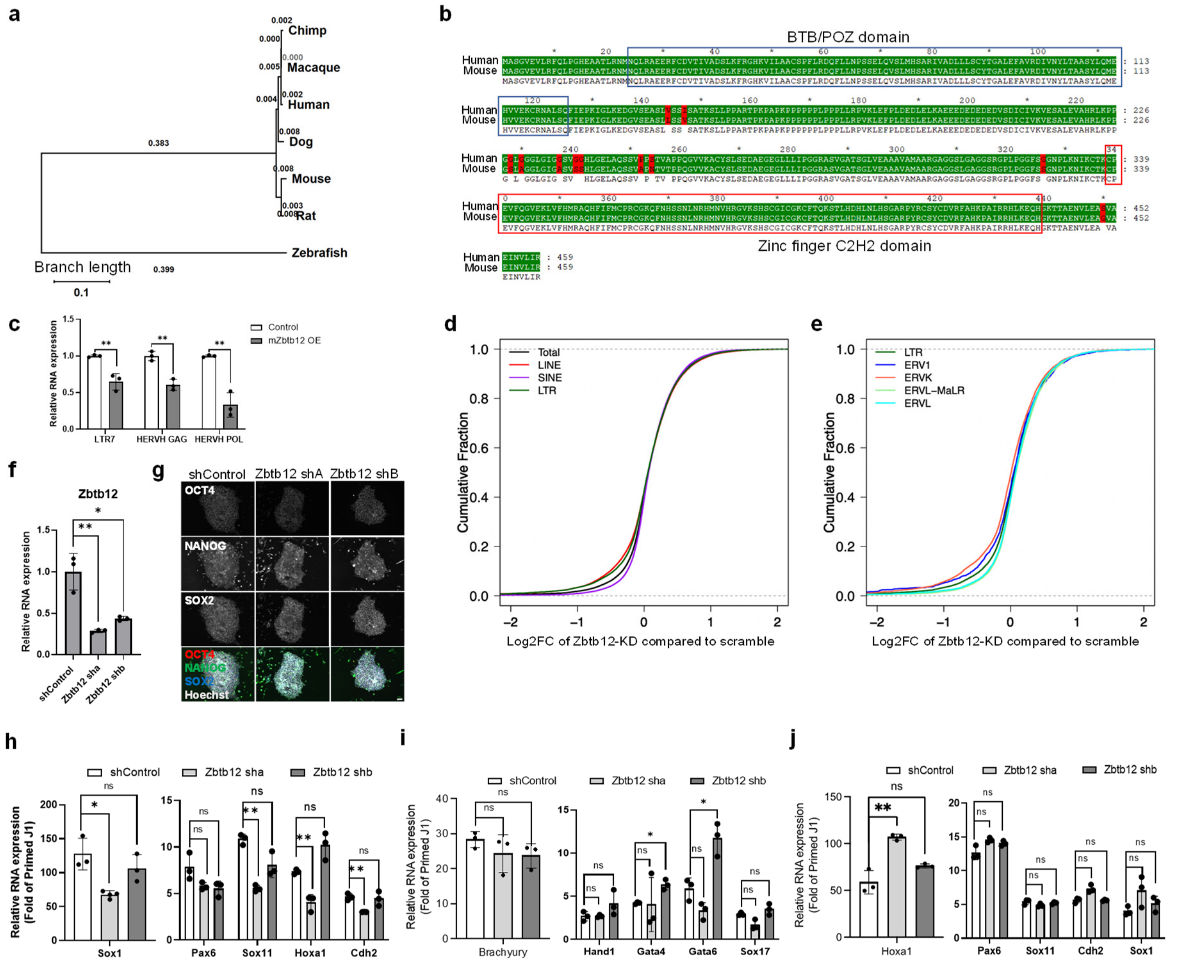

Supplementary Fig. 6 | Evolutionary and functional analysis of mouse Zbtb12. a Neighboring-joining tree of ZBTB12 in vertebrate clades (Phylogenetic tree is constructed using MEGA X based on ZBTB12 protein sequence from different species downloaded from NCBI database). The number above each branch represents branch length. **b** Alignment of ZBTB12 amino acid sequence between human and mouse. A few different residues are observed (highlight red) and located out of the functional domains. **c** qPCR analysis of LTR7, HERVH- GAG and HERVH-POL in H9 hESCs transduced with mouse Zbtb12 lentivirus. Error bars represent mean ± sd (n=3). Student's *t*-test (Holm-Sidak's multiple unpaired) ** p <0.01 compared to Control. **d** Log2 Fold Change of retrotransposon subtypes including LINE, SINE and LTR after Zbtb12 knock-down in J1 mEpiSCs. **e** Log2 Fold Change of LTR, ERV1, ERVK, ERVL-MaLR and ERVL after Zbtb12 KD in J1 mEpiSCs. Statistical analyses (**d**, **e**) can be found in Supplementary Table 10. **f** qPCR analysis of Zbtb12 in J1 mEpiSCs expressing Zbtb12 shRNAs. Error bars represent mean ± sd (n=3). Student's *t*-test (two-tailed unpaired) * p<0.05, ** p <0.01 compared to shControl. **g** Representative images from three independent immunofluorescence assay for OCT4, NANOG and SOX2 in J1 mEpiSCs expressing shControl or Zbtb12 shRNAs. Scale bar, 50 µm. **h, i** qPCR analysis of neuroectoderm (Sox1, Pax6, Sox11, Hoxa1, Cdh2) (**h**) and mesendoderm (Brachyury, Hand1, Gata4, Gata6, Sox17) (**i**) markers in spontaneously differentiated J1 mEpiSCs (day 8) expressing either shControl or Zbtb12 shRNAs. Error bars represent mean ± sd (n=3). Student's *t* test (Holm-Sidak's multiple unpaired or two-tailed unpaired) * p<0.05 and ** p<0.01. ns, non-significant. **j** qPCR analysis of neuroectoderm (Sox1, Pax6, Sox11, Hoxa1, Cdh2) in retinoic acid-differentiated J1 mEpiSCs expressing either shControl or Zbtb12

shRNAs. Error bars represent mean ± sd (n=3). Student's *t*-test (Holm-Sidak's multiple unpaired or two-tailed unpaired) ** $p<0.01$. ns, non-significant. All data points and the exact *p*-values can be found in the Source Data file.

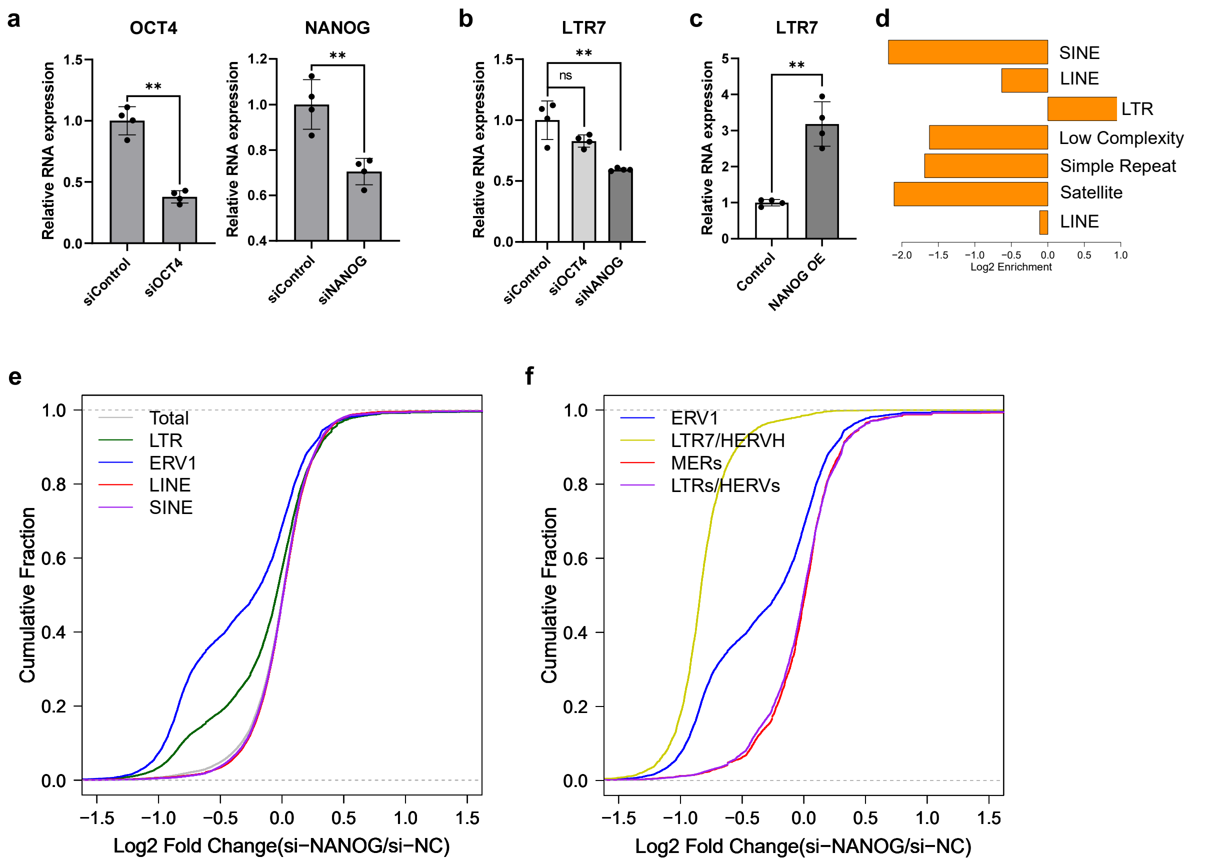

Supplementary Figure 7 | Role of NANOG in HERVH regulation. a qPCR analysis of OCT4 and NANOG in H9 hESCs after siRNAs transfection. Error bars represent mean ± sd (n=4). Student's *t*-test (Holm-Sidak's multiple unpaired) ** p <0.01 compared to siControl. **b** qPCR analysis of LTR7 in H9 hESCs after OCT4 or NANOG KD. Error bars represent mean ± sd (n=4). Student's *t*-test (two-tailed unpaired) ** p <0.01 compared to siControl. ns, non-significant. **c** qPCR analysis of LTR7 in H9 hESCs after NANOG OE. Error bars represent mean ± sd (n=4 for LTR7). Student's *t* test ** p <0.01 compared to Control. **d** Enrichment score of NANOG ChIP-seq data (GSM2816625) in different types of repeat sequences. **e** Log_2 Fold Change of retrotransposon subtypes including LINE (D = 0.0188, p-value < 2.2e-16), SINE (D = 0.013072, p-value = 7.513e-13), LTR (D = 0.15025, p-value < 2.2e-16) and ERV1 (D = 0.357, p- value < 2.2e-16) after NANOG KD (Kolmogorov-Smirnov (KS) test for each subtype compared to total background, n=284397 for Total; n=21267 for LTR; n=82450 for LINE; n=122210 for SINE; n=8223 for ERV1). **f** Log_2 Fold Change of ERV1 (D = 0.32941, p-value < 2.2e-16) family subtypes including LTR7/HERVH (D = 0.85877, p-value < 2.2e-16), MERs and LTRs/HERVs (ERV1 subtypes excluding MERs and LTR7/HERVH) (D = 0.028568, p-value = 0.1183) after NANOG KD (KS test for each subtype compared to MERs subtype, n=8223 for ERV1; n=3085 for LTR7/HERVH; n=for 2529 MERs; n=5138 for LTRs/HERVs). All data points and the exact *p*-values can be found in the Source Data file.

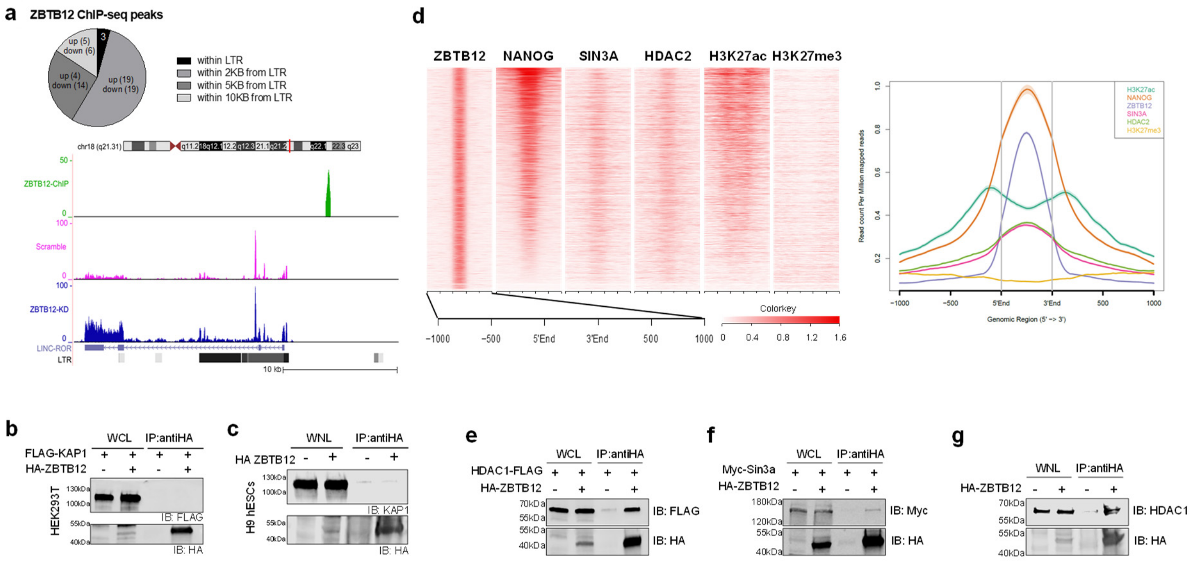

Supplementary Fig. 8 | Molecular feature of ZBTB12-mediated regulation of HERVH. a A pie chart showing distribution of ZBTB12 ChIP-seq peaks associated with full-length HERVH units and an example of ZBTB12 binding locus with ChIP-seq and bulk-RNA seq data. **b** Co- immunoprecipitation assay between FLAG-KAP1 and HA-ZBTB12 in HEK293T. **c** Co- immunoprecipitation assay between endogenous KAP1 and HA-ZBTB12 in H9 hESCs. **d** (left) Comparison of published ChIP-seq peaks of NANOG (GSM2816625), SIN3A (GSM935289), HDAC2 (GSM803345), H3K27ac (GSM663427), and H3K27me3 (GSM433167) to the ZBTB12 binding loci. (right) Histograms showing density of the ChIP-seq reads and their proximity to the ZBTB12 binding loci in hESCs. **e** Co-immunoprecipitation assay between HA-ZBTB12 and HDAC1- FLAG in HEK293T. **f** Co-immunoprecipitation assay between HA-ZBTB12 and Myc-Sin3a in HEK293T. **g** Co-immunoprecipitation assay between HA-ZBTB12 and endogenous HDAC1 in H9 hESCs. Representative images of co-IP blots from three independent experiments (**b**, **c**, **e-g**)

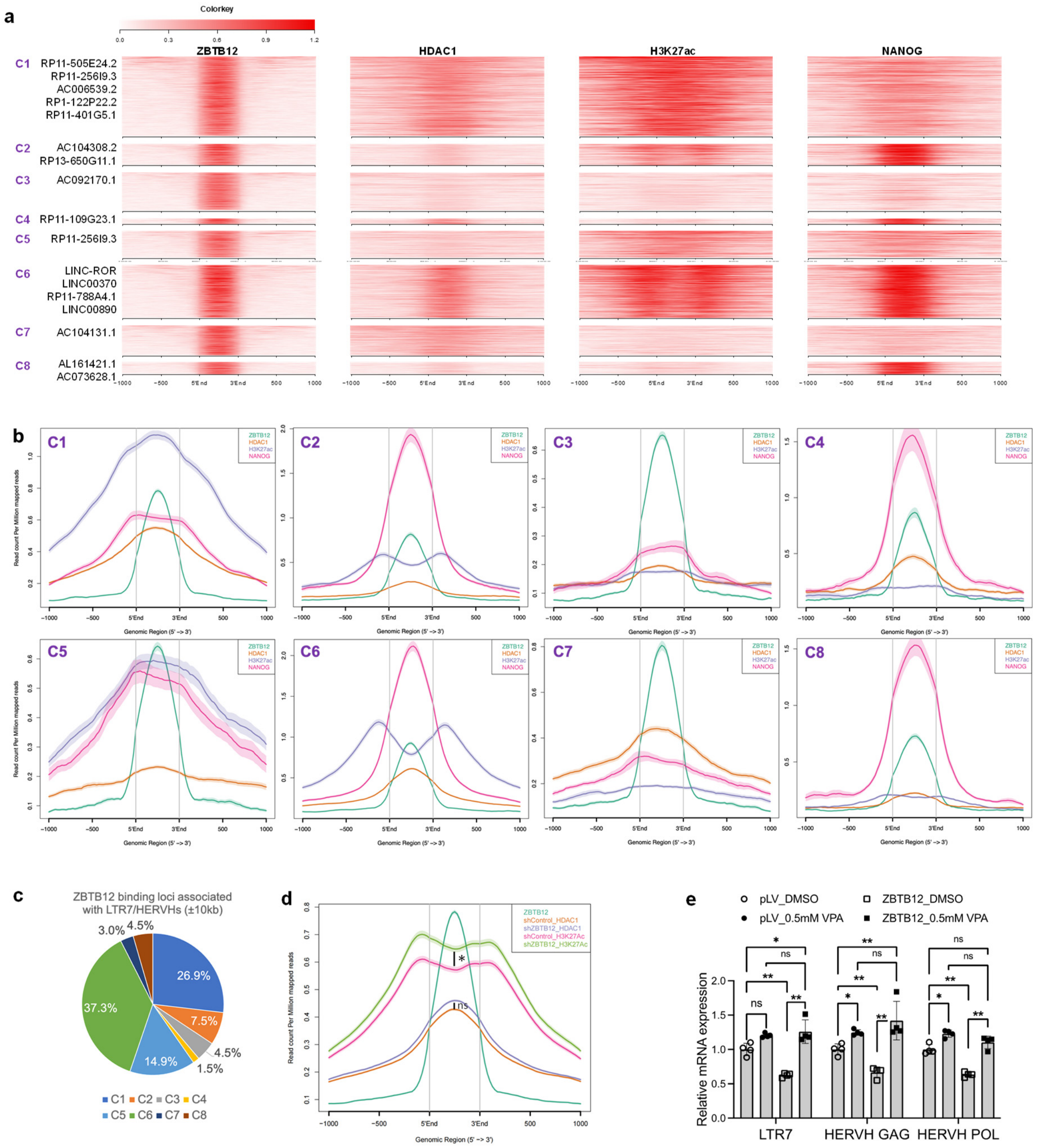

Supplementary Fig. 9 | ChIP-seq analysis of ZBTB12 binding loci. a K-means clustering of ChIP-seq peaks of ZBTB12, HDAC1 and H3K27ac and published ChIP-seq peaks of NANOG (GSM2816625) nearby the ZBTB12 binding loci. C1-C8, Cluster1-Cluster8. Example lncRNAs near the LTR7/HERVHs (10kb within ZBTB12 ChIP-seq peaks) are represented. ChIP-seq peaks and the clustering result can be found in Supplementary Table 13. **b** Individual histograms of each cluster showing density of ZBTB12, HDAC1, H3K27ac and NANOG ChIP-seq reads. **c** A pie chart showing distribution of LTR7-HERVH-associated ZBTB12 binding loci in each cluster. **d** Histograms showing HDAC1 and H3K27ac densities in their proximity to the ZBTB12 binding loci in H9 hESCs expressing either shControl or shZBTB12. Pearson's Chi-squared test: X-squared = 4.4728, df = 1, p-value = 0.03444 for shControl_H3K27ac vs shZBTB12 H3K27ac; X-squared = 0.57376, df = 1, p-value = 0.4488 for shControl HDAC1 vs shZBTB12 HDAC1, * p<0.05. ns, non-significant. n=3621 for shControl_HDAC1, shZBTB12_HDAC1, shControl_H3K27Ac and

shZBTB12_H3K27Ac. **e** qPCR analysis of LTR7, HERVH-GAG, HERVH-POL in vehicle DMSO- or VPA- treated H9 cells either overexpressing empty vector or ZBTB12. Error bars represent mean ± sd (n=4). Two-way ANOVA * p <0.05, ** p <0.01. ns, non-significant. Data points and the exact *p*-values can be found in the Source Data file.

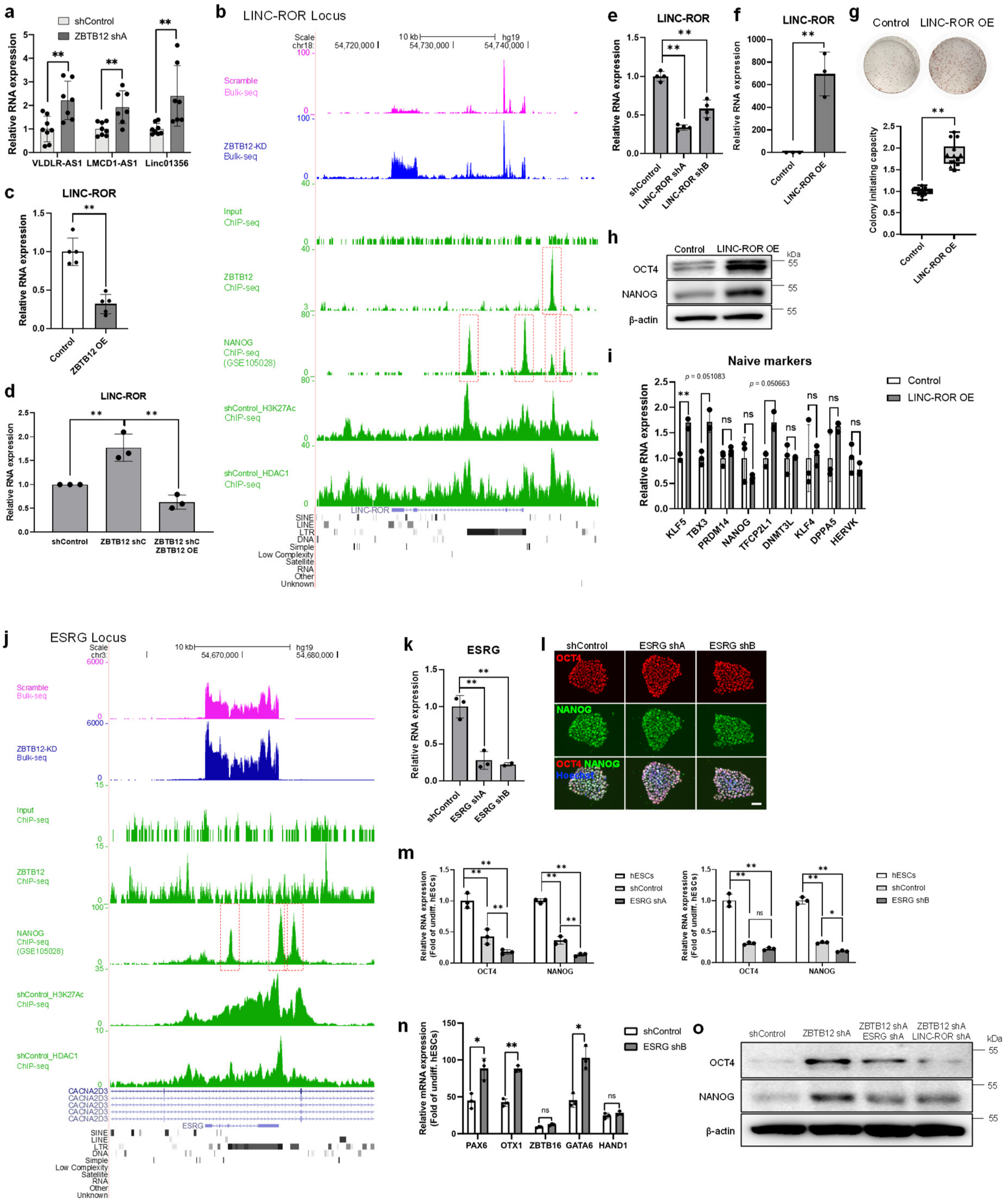

Supplementary Figure 10 | ZBTB12-mediated suppression of LINC-ROR and ESRG facilitates pluripotency exit. a qPCR validation of selected HERVH-associated lncRNAs. Error bars represent mean ± sd (n=8 for shControl, n=7 for ZBTB12 shA). Student's *t* test (two-tailed unpaired) ** p <0.01. **b** A screenshot of LINC-ROR locus displayed on UCSC genome track (http://genome.ucsc.edu) showing bulk RNA-seq (shControl, shZBTB12), ChIP-seq (ZBTB12, NANOG (GSM2816625), H3K27ac, HDAC1) in hESCs. **c** qPCR analysis of LINC-ROR in H9 hESCs after ZBTB12 overexpression. Error bars represent mean ± sd (n=5). Student's *t*-test (Holm-Sidak's multiple unpaired) ** p <0.01. **d** qPCR analysis of LINC-ROR in H9 hESCs expressing ZBTB12 shC with or without ZBTB12

overexpression vector. Error bars represent mean ± sd (n=3). Student's *t*-test (two-tailed unpaired) ** p <0.01. **e** qPCR analysis of LINC-ROR in H9 hESCs expressing LINC-ROR shRNAs. Error bars represent mean ± sd (n=4). Student's *t*-test (two-tailed unpaired) ** p <0.01 compared to shControl. **f** qPCR analysis of LINC-ROR in H9 hESCs after LINC-ROR overexpression. Error bars represent mean ± sd (n=3). Student's *t*-test (Holm-Sidak's multiple unpaired) ** p <0.01. **g** Colony forming assay with H9 hESCs transduced with lentiviral vectors expressing LINC-ROR. Representative AP-stained wells are shown. The number (colony initiating capacity) of colonies is quantified. Data are represented as box-plot with median and min to max with all data points (n=14 from three independent experiments, represented by different point shapes). Center line, median; box limits, upper and lower quartiles; whiskers, max and min; points, all data points. Student's *t*-test (two-tailed unpaired) ** p <0.01. **h** Representative images from three independent western blot assays for OCT4 and NANOG in differentiated (day 7) H9 cells transduced with lentiviral vectors expressing LINC-ROR. **i** qPCR analysis of naïve marker genes in differentiated (day 7) H9 hESCs with LINC-ROR overexpression. Error bars represent mean± sd (n=3). Student's *t*-test (Holm-Sidak's multiple unpaired) ** p <0.01. ns, non-significant. **j** A screenshot of ESRG locus displayed on UCSC genome track (http://genome.ucsc.edu) showing bulk RNA-seq (shControl, shZBTB12), ChIP-seq (ZBTB12, NANOG (GSM2816625), H3K27ac, HDAC1) in hESCs. **k** qPCR analysis of ESRG in H9 expressing ESRG shRNAs. Error bars represent mean ± sd (n=3). Student's *t*-test (two-tailed unpaired) ** p <0.01. **l** Representative images from three independent immunofluorescence assays for OCT4 and NANOG in H9 hESCs after ESRG KD. Scale bar, 25 µm. **m** qPCR analysis of OCT4 and NANOG before (hESCs) and after (shControl and ESRG shRNAs: left, shA; right, shB) differentiation by FGF2 and TGFβ deprivation. Error bars represent mean ± sd (n=3). Two-way ANOVA, *p<0.05 and ** p <0.01, ns, non-significant. **n** qPCR analysis of neuroectoderm (PAX6, OTX1, ZBTB16) and mesendoderm (GATA6, HAND1) markers in differentiated (day 8) H9 cells transduced with lentiviral vectors expressing shControl or ESRG shB. Error bars represent mean ± sd (n=3). Student's *t*-test (Holm-Sidak's multiple unpaired) * p <0.05 and ** p <0.01. ns, non-significant. **o** Representative images from three independent western blot assays for OCT4 and NANOG in differentiated (day 7) H9 cells transduced with lentiviral vectors expressing ZBTB12 shA in combination with ESRG shA or LINC-ROR shA. Dotted red boxes in ZBTB12 ChIP-seq and NANOG ChIP-seq tracks indicate significant peaks (**b**, **j**).

Supplementary Table 1 | Stage specific transcription factor motif analysis from nanoCAGE data. Motif analysis for stage specific peaks (n=2335 for D0; n=2345 for D5; n=863 for D15; n=1130 for D23); Motif was predicted compared to background sequence at each stage by HOMER software (findMotifsGenome.pl peaks.txt hg19 output -size -250,50). P value of each predicted transcription factor (tf) was corrected by Benjamini-Hochberg method.

**Stage specific TF motif analysis**				
**TF**	**pval**			
	**day0**	**day5**	**day15**	**day23**
JUND	1.00E+00	1.00E-02	1.00E+00	1.00E-08
RFX3	1.00E+00	1.00E+00	1.00E-03	1.00E-08
MAZ	1.00E-23	1.00E-08	1.00E-04	1.00E-07
KLF14	1.00E-20	1.00E-05	1.00E+00	1.00E-07
RFX2	1.00E+00	1.00E+00	1.00E-03	1.00E-07
SP1	1.00E-33	1.00E-17	1.00E-05	1.00E-06
ATF2	1.00E+00	1.00E+00	1.00E+00	1.00E-06
ELK4	1.00E-04	1.00E-07	1.00E-05	1.00E-04
FLI1	1.00E-02	1.00E-04	1.00E-02	1.00E-04
ATF1	1.00E+00	1.00E+00	1.00E-01	1.00E-04
ATF7	1.00E+00	1.00E+00	1.00E+00	1.00E-04
KLF5	1.00E-24	1.00E-07	1.00E+00	1.00E-03
KLF9	1.00E-11	1.00E-15	1.00E-02	1.00E-03
ZNF467	1.00E-10	1.00E-05	1.00E+00	1.00E-03
ETS1	1.00E-06	1.00E-04	1.00E-03	1.00E-03
ELK1	1.00E-05	1.00E-08	1.00E-03	1.00E-03
JUN	1.00E+00	1.00E+00	1.00E+00	1.00E-03
KLF10	1.00E-14	1.00E-03	1.00E+00	1.00E-02
MYB	1.00E-10	1.00E-04	1.00E-01	1.00E-02
EHF	1.00E-07	1.00E-03	1.00E-01	1.00E-02
ELF1	1.00E-06	1.00E-05	1.00E-05	1.00E-02
NRF1	1.00E-03	1.00E-07	1.00E-04	1.00E-02
ETV1	1.00E-01	1.00E-03	1.00E-02	1.00E-02
ZNF263	1.00E+00	1.00E-04	1.00E-01	1.00E-02
KLF4	1.00E-25	1.00E-02	1.00E+00	1.00E-01
MYBL2	1.00E-08	1.00E-01	1.00E-01	1.00E-01
SOX3	1.00E-07	1.00E+00	1.00E+00	1.00E-01
NANOG	1.00E-06	1.00E-06	1.00E-01	1.00E-01
EGR1	1.00E-06	1.00E-01	1.00E+00	1.00E-01
NF1	1.00E-04	1.00E-01	1.00E+00	1.00E-01

ARNT	1.00E-03	1.00E-01	1.00E+00	1.00E-01
NR4A1	1.00E-03	1.00E+00	1.00E-01	1.00E-01
GABPA	1.00E-01	1.00E-04	1.00E-01	1.00E-01
DMC1	1.00E-01	1.00E-03	1.00E-01	1.00E-01
KLF1	1.00E-23	1.00E+00	1.00E+00	1.00E+00
MAFF	1.00E-23	1.00E+00	1.00E+00	1.00E+00
TEAD2	1.00E-18	1.00E+00	1.00E+00	1.00E+00
ZIC1	1.00E-16	1.00E+00	1.00E+00	1.00E+00
HOXB13	1.00E-14	1.00E+00	1.00E+00	1.00E+00
TEAD4	1.00E-12	1.00E+00	1.00E+00	1.00E+00
IRF8	1.00E-10	1.00E+00	1.00E+00	1.00E+00
BHLHE40	1.00E-09	1.00E+00	1.00E+00	1.00E+00
MYC	1.00E-08	1.00E+00	1.00E+00	1.00E+00
GATA4	1.00E-08	1.00E+00	1.00E+00	1.00E+00
RUNX2	1.00E-07	1.00E-01	1.00E+00	1.00E+00
MAX	1.00E-07	1.00E+00	1.00E+00	1.00E+00
MAFA	1.00E-06	1.00E-01	1.00E-01	1.00E+00
EOMES	1.00E-06	1.00E+00	1.00E+00	1.00E+00
GATA2	1.00E-06	1.00E+00	1.00E+00	1.00E+00
GATA3	1.00E-06	1.00E+00	1.00E+00	1.00E+00
USF1	1.00E-06	1.00E+00	1.00E+00	1.00E+00
CLOCK	1.00E-05	1.00E+00	1.00E+00	1.00E+00
PTF1A	1.00E-05	1.00E+00	1.00E+00	1.00E+00
TCF12	1.00E-04	1.00E+00	1.00E+00	1.00E+00
MITF	1.00E-04	1.00E+00	1.00E+00	1.00E+00
MYCN	1.00E-04	1.00E+00	1.00E+00	1.00E+00
ZBTB12	1.00E-04	1.00E+00	1.00E+00	1.00E+00
POU3F3	1.00E-03	1.00E+00	1.00E+00	1.00E+00
POU5F1	1.00E-03	1.00E+00	1.00E-01	1.00E+00
SOX2	1.00E-03	1.00E+00	1.00E+00	1.00E+00
TGIF2	1.00E-03	1.00E+00	1.00E-01	1.00E+00
ZNF322	1.00E-03	1.00E+00	1.00E+00	1.00E+00
EGR2	1.00E-02	1.00E-04	1.00E+00	1.00E+00
E2F4	1.00E-02	1.00E-03	1.00E-01	1.00E+00

Supplementary Table 2 | Plasmid DNA constructs used in this study

Recombinant DNA	Source	More info
pLV-EF1A-IRES-puro	Vectorbuilder	https://en.vectorbuilder.com
pLV-EF1A-HA-ZBTB12-IRES-puro	Vectorbuilder	https://en.vectorbuilder.com
pLV-EF1A-ZBTB12-FLAG-IRES-puro	Vectorbuilder	https://en.vectorbuilder.com
pLV-EF1A-HA-mZbtb12-IRES-puro	Vectorbuilder	https://en.vectorbuilder.com
pSin-EF2-Nanog-Pur	Addgene	16578
pCS2+MT-mSin3A	Addgene	30452
HDAC1 Flag	Addgene	13820
pLKO.1-scramble shRNA	Addgene	1864
pLKO.1-GFP shRNA	Addgene	12273
pLKO.1-ZBTB12 shA	SigmaAldrich	TRCN0000164972
pLKO.1-ZBTB12 shB	SigmaAldrich	TRCN0000165491
pLV-U6-scramble shRNA-EGFP:T2A:Bsd	Vectorbuilder	Target sequence: CAACAAGATGAAGAGCACCAA
pLV-U6-ZBTB12 shC-EGFP:T2A:Bsd (targeting 3'UTR)	Vectorbuilder	Target sequence: GATGATTCCTTGGAGAGACTT
pLKO.1-LINCROR shA	Addgene	45764
pLKO.1-LINCROR shB	Addgene	45765
pLV-U6-LINCROR shA-EGFP:T2A:Bsd	Vectorbuilder	Target sequence: AAGCCTGAGAGTTGGCATGAA
pLV-EF1A-lincROR-mPGK-puro	In house cloning	LincROR is amplified from pBABE-LINCROR (Addgene #45763)
pLV-U6-scramble shRNA-EGFP:T2A:Puro	Vectorbuilder	Target sequence: CAACAAGATGAAGAGCACCAA
pLV-U6-ESRG shA-EGFP:T2A:Puro	Vectorbuilder	Target sequence: GCTTCGCACCATTGAATAAAC
pLV-U6-ESRG shB-EGFP:T2A:Puro	Vectorbuilder	Target sequence: GGGTCCATCTTCAACCAAACT
pLV-EGFP:T2A:Puro-U6>mZbtb12 sha	Vectorbuilder	Target sequence:GAACGCCCTTAGCCAGTTCAT
pLV-EGFP:T2A:Puro-U6>mZbtb12 shb	Vectorbuilder	Target sequence:TCTGACATCTGCATCGTCAAA
pLV-LacI:T2A:Puro-U6/2xLacO>scramble shRNA	Vectorbuilder	Target sequence:CCTAAGGTTAAGTCGCCCTCG
pLV-LacI:T2A:Puro-U6/2xLacO>hZBTB12 shB	Vectorbuilder	Target sequence:GATTAACGTCCTCATCCGCTA
pMD2.G	Addgene	12259
psPAX2	Addgene	12260

Supplementary Table 3 | Primers used in this study

Targets	(Forward) 5'-3'	(Reverse) 5'-3'
ZBTB12	GGCAAGCAGTTCAACCACAG	CAGGTTGAGGTGGTCGTGAA
OCT4 (POU5F1)	GAGAAGGAGAAGCTGGAGCA	AATAGAACCCCCAGGGTGAG
NANOG	GATTTGTGGGCCTGAAGAAA	CAGATCCATGGAGGAAGGAA
SOX2	CATGGACAGTTACGCGCACAT	AGTTGTACTGCAGGGCGCTCA
PAX6	GGCTAGCGAAAAGCAACAGA	TGGTATTCTCTCCCCCTCCT
OTX1	CACCGCAGCAGCCTCTTATCC	TGCATGGGCGCTAGGTATGAG
ZBTB16	AAGCGGTTCCTGGATAGTTTG	CATGTCAGTGCCAGTATGGGT
GATA6	AGGGCTCGGTGAGTCCAAT	CGCTGCTGGTGAATAAAAAGGA
HAND1	TCAGCCTTGCCCGGACTCTC	AGGTTCATGTTGGAGCGGCTAC
SOX17	CGCTTTCATGGTGTGGGCTAAG	CGCTCTGCCTCCTCCACGAAG
LTR7	GCTGTCTGTGAAGCTTTGCG	ACTATTCCTTTGCACCCTTAATCCC
HERVH-GAG	ACGCTTTACAGCCCTAGACC	GTCGGGAGCAGATTGGGTAA
HERVH-POL	CGCCCTTCTTCCCAATCCAA	GCCAAGGAGGGAGTAGAGGT
LINC-ROR	TATAGTTCTTCCAGGTCTCAGG	CTTTCGAGGTTATCAGGGTG
ESRG	TGGGATGGAGCCATAGAAGT	TGGGTCTTTCAAGAAGTTCCTC
VLDLR-AS1	AAGCTAAAGACATGCCTTAAAGGATGA	TCTGAAGAAACAGAGAAAGTCATCTTTTCCTC
LMCD1-AS1	TCCCGCGATCCATTCAGGT	GGGCTTGGCTTATGGCAAGTATC
Linc01356	CATCACAGATGCCCAGCTTTGG	AGGATGAGCCAGGAGAAGGAAT
KLF4	GATTTGTGGGCCTGAAGAAA	CAGATCCATGGAGGAAGGAA
KLF5	GTCAGCCTCCTCTTCGTCGTC	GCGGACTCCCTGCCATAGA
TBX3	TAAACTGGCAATTCACAATCC	CAACCAGGGTAATCGCAGTA
DPPA5	CCGGTTCCACATTGTAAGAG	GTATGCAGTCACAGCGATGAA
PRDM14	AAGCCATGAATGCCCTCGAA	CTCTAGCTCTGGCCACAACC
TFCP2L1	AAGTACACCCCCTGTGTGGA	CAGAGTGGACTCGCATGTGT
DNMT3L	GTGAGGCCAAAGATGACCATT	GGAAGATGGCGTGGTACACA
HERVK	CTCTCAAGCTCCGTTTCACC	GTACAGGAAGAGGGCATCCA
GAPDH	GAAGGTGAAGGTCGGAGTC	GAAGATGGTGATGGGATTTC
mouse Zbtb12	AGGCTAGCCTCCTAAGCAGT	TAGGTCCTCATCCAGCGGAA
mouse Pax6	GAACAGTCACAGCGGAGTGA	CCGCTGTGAGCTAGCTGTAC
mouse Sox1	TAAGTGCGTGTGCTTTTGCC	AAGAGAGGGCCTGGAGCATA
mouse Sox11	GAGTTCCCCGACTACTGCAC	TTTATCCTGACCGCCACGAC
mouse Hoxa1	GGAAGCAGACCCACCAAGAA	TGAGCTGCTTGGTGGTGAAA
mouse Cdh2	AGCCCGGTTTCACTTGAGAG	CATCCGCATCAATGGCAGTG

mouse Gata6	GCGCCTCCTCTCTCCTTTTT	GCGCTACTCCAACCTGACTT
mouse Hand1	CAAGCGGAAAAGGGAGTTGC	GCTTGGTCTTCTCCAGTCCC
mouse Brachyury	AACTGGTCTAGCCTCGGAGT	TGGGGTGATGGTACCATTGC
mouse Gata4	CTGTGCCAACTGCCAGACTA	TTTGAATCCCCTCCTTCCGC
mouse Sox17	GCTCCAGTCTCGGACTATGC	CCGTAGTACAGGTGCAGAGC

Supplementary Table 4 | Antibodies and reagents used in this study

REAGENT	SOURCE	IDENTIFIER	Information	
**Antibodies**			**dilution**	**clone name**
Anti-ZBTB12	Novus Biologicals	H00221527-B01P	ICC, 1:100	Polyclonal
Anti-OCT3/4	Santa Cruz Biotechnology	sc-5279	ICC, WB 1:200	C10 monoclonal
Anti-NANOG	R&D Systems	AF1997	ICC, WB 1:1000	Polyclonal
Anti-PolII	Sigma-Aldrich	05-623	5µg for ChIP-seq	CTD4H8
Anti-FLAG (for chromatin immunoprecipitation)	Cell Signaling Technologies	14793	5µg for ChIP-seq	D6W5B
Pierce™ Anti-HA Magnetic Beads (for immunoprecipitation)	ThermoFisher Scientific	88836	NA	2-2.2.14
Anti-HA tag	Cell Signaling Technologies	3724	WB, 1:1000; ICC, 1:200	C29F4
Anti-myc tag	Cell Signaling Technologies	2272	WB, 1:1000	Polyclonal
Anti-HDAC1	Cell Signaling Technologies	34589	WB, 1:1000	D5C6U
Anti-KAP1	Abcam	Ab10484	WB, 1:500	Polyclonal
Anti-β-actin	Santa Cruz Biotechnology	sc-47778	WB, 1:5,000	C4 monoclonal
Anti-SOX2	Sigma	AB5603	ICC , 1:1000	Polyclonal
anti-HDAC1 (for chromatin immunoprecipitation)	Active Motif	40967	4 µg	Polyclonal
anti-H3K27ac (for chromatin immunoprecipitation)	Active Motif	39133	4 µg	Polyclonal
**Chemicals, Peptides, and Recombinant Proteins**				
mTeSR1	Stem Cell Technologies	85875		
TeSR-E8	Stem Cell Technologies	5990		

DMEM/F-12, GlutaMAX™ supplement	Life Technologies	10565-018		
DMEM(high glucose)	ThermoFisher Scientific	11995-065		
DMEM/F-12	ThermoFisher Scientific	11320-033		
Knockout Replacement Serum	Life Technologies	10828-028		
Matrigel hESC-Qualified Matrix	Fisher	08-774-552		
StemPro Accutase	Life Technologies	A1110501		
ReLeSR	Stem Cell Technologies	5872		
MEM Non-essential amino acids (100X)	Life Technologies	11140-050		
Y-27632	Tocris/Fisher	1254/10		
2-Mercaptoethanol	ThermoFisher Scientific	31350-010		
DMEM	Life Technologies	11965092		
Fetal bovine serum	Life Technologies	10082-147		
Penicillin-Streptomycin	Life Technologies	15140122		
0.25% Trypsin-EDTA (1X), Phenol Red	Life Technologies	25200-056		
Valproic acid	Sigma	PHR1061-1G		
TRIzol™ Reagent	ThermoFisher Scientific	15596018		
2X Laemmli Sample Buffer	BioRad	1610737		
Human leukemia inhibitory factor	Millipore	LIF1010		
Dorsomorphin	Tocris	3093		
SB431542	PEPROTECH	3014193		
BIO	Tocris	3194		
PD0325901	Sigma	PZ0162		
Senexin A (CDK8/19 inhibitor)	Tocris	4875		

IPTG	ThermoFisher Scientific	AM9462		
Recombinant Human/Murine/Rat Activin A	PEPROTECH	120-14E		
Recobinant Murine FGF-basic	PEPROTECH	450-33		
Gentamicin Reagent	ThermoFisher Scientific	15750-060		
GlutaMAX(100X)	ThermoFisher Scientific	35050-061		
Retinoic acid	Sigma	R2625-50MG		
Recombinant Human BMP4 protein	R&D Systems	314-BP-010		
