# Supplementary material for: ZBTB12 is a molecular barrier to dedifferentiation in human pluripotent stem cells
Source: Nat Commun. 2023 Feb 9;14:632. doi: 10.1038/s41467-023-36178-9 (PMC9911396; doi:10.1038/s41467-023-36178-9)
Supplement: Supplementary file 3 — Description of Supplementary Data [file 41467_2023_36178_MOESM3_ESM.docx]

**Description of Additional Supplementary Files**

**ZBTB12 is a molecular barrier to dedifferentiation in human pluripotent stem cells**

**Contents:**

Supplementary DATA 1-14

**SUPPLEMENTARY DATA**

**Supplementary Data 1**

Stage specific nanoCAGE peaks during neural differentiation of H9 hESCs

**Supplementary Data 2**

Differentially expressed genes in Day0 shZBTB12 (versus Day0 shCtrl from Fig. 2)

MAST algorithm is used to statistically determine DEGs, with Benjamini-Hochberg-corrected p values <0.05.

**Supplementary Data 3**

Differentially expressed genes in Cluster 1 (versus Cluster 0 and 2 from Fig. 3a)

MAST algorithm is used to statistically determine DEGs, with Benjamini-Hochberg-corrected p values <0.05.

**Supplementary Data 4**

WGCNA module gene lists (brown, blue and turquoise from Supplementary Fig. 4)

**Supplementary Data 5**

ZBTB12-FLAG ChIP-seq peaks

**Supplementary Data 6**

ZBTB12-FLAG ChIP-seq motif analysis

**Supplementary Data 7**

Target information of qPCR primers for LTR7, HERVH GAG and HERVH POL

**Supplementary Data 8**

Differentially expressed coding genes (bulk RNA-seq) after ZBTB12 KD

Gene expression of 3 biological samples for ZBTB12-KD and Scramble were compared with DESeq software using Wald significance tests. P value was corrected using Benjamini and Hochberg method.

**Supplementary Data 9**

Kolmogorov-Smirnov test for Supplementary Fig. 6d, e

**Supplementary Data 10**

Expression of ERV1 elements in H9 hESCs transduced or transfected with shControl, shZBTB12, siControl and siNANOG (Fig. 4i)

**Supplementary Data 11**

ZBTB12-FLAG ChIP-seq peaks associated with full length LTR7/HERVH

**Supplementary Data 12**

ChIP-seq peaks (HDAC1, H3K27ac) and k-means clustering

**Supplementary Data 13**

280 lncRNAs overlapped with LTR7/HERVH loci

**Supplementary Data 14**

56 HERVH-overlapping lncRNAs detected in our RNA-seq data with counts per million (CPM) higher than one
