# Supplementary material for: ZBTB12 is a molecular barrier to dedifferentiation in human pluripotent stem cells
Source: Nat Commun. 2023 Feb 9;14:632. doi: 10.1038/s41467-023-36178-9 (PMC9911396; doi:10.1038/s41467-023-36178-9)
Supplement: Supplementary file 18 — Reporting Summary [file 41467_2023_36178_MOESM18_ESM.pdf]

nature portfolio

Corresponding author(s): Kenneth S. Kosik
Jiwon Jang

Last updated by author(s): Jan 1, 2023

Reporting Summary

Nature Portfolio wishes to improve the reproducibility of the work that we publish. This form provides structure for consistency and transparency in reporting. For further information on Nature Portfolio policies, see our Editorial Policies and the Editorial Policy Checklist.

Statistics

For all statistical analyses, confirm that the following items are present in the figure legend, table legend, main text, or Methods section.

n/a	Confirmed	
[ ]	[x]	The exact sample size (*n*) for each experimental group/condition, given as a discrete number and unit of measurement
[ ]	[x]	A statement on whether measurements were taken from distinct samples or whether the same sample was measured repeatedly
[ ]	[x]	The statistical test(s) used AND whether they are one- or two-sided<br>*Only common tests should be described solely by name; describe more complex techniques in the Methods section.*
[x]	[ ]	A description of all covariates tested
[x]	[ ]	A description of any assumptions or corrections, such as tests of normality and adjustment for multiple comparisons
[ ]	[x]	A full description of the statistical parameters including central tendency (e.g. means) or other basic estimates (e.g. regression coefficient) AND variation (e.g. standard deviation) or associated estimates of uncertainty (e.g. confidence intervals)
[ ]	[x]	For null hypothesis testing, the test statistic (e.g. *F*, *t*, *r*) with confidence intervals, effect sizes, degrees of freedom and *P* value noted<br>*Give P values as exact values whenever suitable.*
[x]	[ ]	For Bayesian analysis, information on the choice of priors and Markov chain Monte Carlo settings
[x]	[ ]	For hierarchical and complex designs, identification of the appropriate level for tests and full reporting of outcomes
[x]	[ ]	Estimates of effect sizes (e.g. Cohen's *d*, Pearson's *r*), indicating how they were calculated

Our web collection on statistics for biologists contains articles on many of the points above.

Software and code

Policy information about availability of computer code

Data collection	Confocal images were taken by Leica TCS SP8 confocal microscope (Leica). Realtime PCR result were collected by QuantStudio 12K Flex Real-Time PCR System (ThermoFisher Scientific). Western blot results were visualized with LI-COR Odyssey Imaging System (LI-COR).
Data analysis	Sequencing data sets were processed and analyzed using the following tools:<br>nanoCAGE data, ChIP-seq data and Bulk RNA-seq data:<br>bwa software(version 0.7.13)<br>samtools (version: 0.1.16)<br>HOMER (version3.12)<br>R(version 3.6.3)<br>R package ggplot2_3.3.6<br>R package clustepofiler (version3.12)<br>Mathematic 11.3<br>Python(version2.7.10)<br>Python package HT seq(version0.7.2)<br>Bedtools(version2.16.1)<br>MACS (v2)<br>TopHat(version2.1.1)<br>ngsplot(version 2.63)

scRNA-seq data:
Cell Ranger toolkit (version 3.1.0)
Seurat(version 3.1.5)
CytoTRACE(http://cytotrace.stanford.edu)
Slingshot (version 1.4.0)
monocle3 (version 0.2.1)
WGCNA(version 1.70)

Evolutionary anaylsis were processed and analyzed using the following tools of parameters:
MEGA X
Genedoc (version2.7.0)

For manuscripts utilizing custom algorithms or software that are central to the research but not yet described in published literature, software must be made available to editors and reviewers. We strongly encourage code deposition in a community repository (e.g. GitHub). See the Nature Portfolio guidelines for submitting code & software for further information.

Data

Policy information about availability of data

All manuscripts must include a data availability statement. This statement should provide the following information, where applicable:
- Accession codes, unique identifiers, or web links for publicly available datasets
- A description of any restrictions on data availability
- For clinical datasets or third party data, please ensure that the statement adheres to our policy

Data are available in the main text, supplementary materials, and Gene Expression Omnibus (GSE167052, GSE118946, and GSE205342)

Human research participants

Policy information about studies involving human research participants and Sex and Gender in Research.

Reporting on sex and gender	Not applicable
Population characteristics	Not applicable
Recruitment	Not applicable
Ethics oversight	Not applicable

Note that full information on the approval of the study protocol must also be provided in the manuscript.

Field-specific reporting

Please select the one below that is the best fit for your research. If you are not sure, read the appropriate sections before making your selection.

[x] Life sciences [ ] Behavioural & social sciences [ ] Ecological, evolutionary & environmental sciences

For a reference copy of the document with all sections, see nature.com/documents/nr-reporting-summary-flat.pdf

Life sciences study design

All studies must disclose on these points even when the disclosure is negative.

Sample size	Preliminary experiments were performed when possible to determine requirements for sample size. Sample size sufficiency was determined by preliminary data or discussion. For statistical significance, the sample size was always independently performed three or more times(except for a few supplementary data with two independent experiments).
Data exclusions	No data were excluded from the analysis.
Replication	All experiments were replicated or performed independently for at least three times(except for a few supplementary data with two independent experiments), and all attempts at replication were successful.
Randomization	Randomization was not relevant to our study design.
Blinding	Blinding was not possible in this study because the same investigators performed cell culture and biochemical analyses.

Reporting for specific materials, systems and methods

We require information from authors about some types of materials, experimental systems and methods used in many studies. Here, indicate whether each material, system or method listed is relevant to your study. If you are not sure if a list item applies to your research, read the appropriate section before selecting a response.

Materials & experimental systems

n/a	Involved in the study
☐	☒ Antibodies
☐	☒ Eukaryotic cell lines
☒	☐ Palaeontology and archaeology
☒	☐ Animals and other organisms
☒	☐ Clinical data
☒	☐ Dual use research of concern

Methods

n/a	Involved in the study
☐	☒ ChIP-seq
☒	☐ Flow cytometry
☒	☐ MRI-based neuroimaging

Antibodies

Antibodies used

Anti-ZBTB12, Novus Biologicals, H00221527-B01P (Polyclonal)
Anti-OCT3/4, Santacruz Biotechnology, sc-5279 (C10 monoclonal)
Anti-NANOG, R&D Systems, AF1997 (Polyclonal)
Anti-SOX2, Sigma, AB5603 (Polyclonal)
Anti-PolII (for chromatin immunoprecipitation), sigma-Aldrich, 05-623(CTD4H8, monoclonal)
Anti-FLAG (for chromatin immunoprecipitation), Cell Signaling Technologies, 14793(monoclonal)
Anti-HA tag, Cell Signaling Technologies, 3724(C29F4,monoclonal)
Anti-myc tag, Cell Signaling Tehnologies, 2272(Polyclonal)
Anti-HDAC1, Cell Signaling Technologies, 34589(D5C6U, monoclonal)
Anti-KAP1, Abcam, Ab10484(Polyclonal)
Anti-β-actin, Santa Cruz Biotechnology, sc-47778(C4, monoclonal)
Anti-HDAC1, Active Motif, 40967(Polyclonal)
Anti-H3K27ac, Active Motif, 39133(Polyclonal)

Validation

-Anti-ZBTB12, Novus Biologicals, H00221527-B01P: ZBTB12 Knockdown using two independent shRNAs led to a reduced fluorescence signal shown by immunofluorescent staining(Supplementary Figure 2d). The antibody guarantee covers the use of the antibody for WB and IF applications. Species reactivity: Human

-Anti-OCT3/4, Santa Cruz Biotechnology, sc-5279: hESCs differentiation led to a reduced fluorescence signal shown by immunofluorescent staining(Figure 1b, 1e). The antibody guarantee covers the use of the antibody for WB and IF applications. No cross-reactivity may occur with Oct-3/4 isoform B. Species reactivity: Mouse, Rat and Human

-Anti-NANOG, R&D Systems, AF1997: hESCs differetiation led to a reduced fluorescence signal shown by immunofluorescent staining (Figure 1b, 1e). The antibody guarantee covers the use of the antibody for WB and IF applications. Species reactivity: Human

-Anti-SOX2, Sigma, AB5603: hESCs differentiation led to a reduced fluorescence signal shown by immunofluorescent staining(Data not included). The antibody guarantee covers the use of the antibody for WB and IF applications. Species reactivity: Human, Mouse.

-Anti-PolII (for chromatin immunoprecipitation), sigma-Aldrich, 05-623: The antibody guarantee covers the use of the antibody for ChIP-application and WB. Species reactivity: Saccharomyces cerevisiae, Human, Mouse, Rat

- Anti-FLAG (for chromatin immunoprecipitation), Cell Signaling Technologies, 14793: The antibody guarantee covers the use of the antibody for WB, IF, flow cytometry and ChIP application. Species reactivity: all species expected

-Anti-HA tag, Cell Signaling Technologies, 3724: The antibody was validated by staining H9 hESCs expressing HA ZBTB12 (Supplementary Figure 2b). The antibody guarantee covers the use of the antibody for WB, IP, IF and flow cytometry

-Anti-myc tag, Cell Signaling Tehnologies, 2272: The antibody guarantee covers the use of the antibody for WB, IF and flow cytometry. The antibody was validated by staining HEK293T cells expressing Myc-Sin3a(Supplementary Figure 8f). Species reactivity: all species expected

-Anti-HDAC1, Cell Signaling Technologies, 34589. Anti-HDAC1 detects endogenous levels of total HDAC1 protein. The antibody guarantee covers the use of the antibody for WB, IP and IF. Species reactivity: human, rat, mouse and monkey.

-Anti-KAP1, Abcam, Ab10484: The antibody guarantee covers the use of the antibody for WB and IP. Species reactivity: Mouse, Human

-Anti-β-actin, Santa Cruz Biotechnology, sc-47778: The antibody guarantee covers the use of the antibody for WB, IP, IF and ELISA.

Species reactivity: mouse, rat, human, avian, bovine, canine, porcine, rabbit, Dictyostelium discoideum and Physarum polycephalum. Cross-reactivity may occur with all six known isoforms of Actin in higher vertebrates (including cytoplasmic β- and γ- Actin isoforms, skeletal, cardiac, and vascular α-Actin isoforms, and enteric γ-Actin isoform).

-Anti-HDAC1, Active Motif, 40967: The antibody guarantee covers the use of the antibody for ChIP and WB. Species reactivity: Human, Mouse, Rat

-Anti-H3K27ac, Active Motif, 39133: The antibody guarantee covers the use of the antibody for ChIP, IF and WB. Species reactivity: Budding Yeast, Human, Wide Range Predicted.

Eukaryotic cell lines

Policy information about cell lines and Sex and Gender in Research

Cell line source(s)	H1, H9 hESCs and IMR90 hiPSCs were purchased from WiCell.<br>J1 mEpiSCs were provided by Hyuk-Jin Cha at Seoul National University.<br>HEK293T was purchased from ATCC.
Authentication	Cell lines were authenticated by short tandem repeat analysis and/or in vitro differentiation.
Mycoplasma contamination	Mycoplasma contamination was routinely checked and negative results were obtained.
Commonly misidentified lines<br>(See ICLAC register)	No commonly misidentified cell lines were used in the study.

ChIP-seq

Data deposition

☒ Confirm that both raw and final processed data have been deposited in a public database such as GEO.

☒ Confirm that you have deposited or provided access to graph files (e.g. BED files) for the called peaks.

Data access links
May remain private before publication.

https://www.ncbi.nlm.nih.gov/geo/query/acc.cgi
token for reviewer's access: uxktieqwpzqvxil(GSE118946), oxulismrdebbqt(GSE205342)

Files in database submission

Rawdata:
GSM3351655 Input_DNA
GSM3351656 PolII_ChIPSeq
GSM3351657 ZBTB12_Flag_ChIPSeq

GSM6210722 Pooled input
GSM6210718 anti-H3K27ac, ChIP, H9, shGFP
GSM6210719 anti-H3K27ac, ChIP, H9, shZBTB12
GSM6210720 anti-HDAC1, ChIP, H9, shGFP
GSM6210721 anti-HDAC1, ChIP, H9, shZBTB12
Processed file:
GSE118946_Input.bw
GSE118946_PolII.bw
GSE118946_ZBTB12_flag.bw
GSE118946_ZBTB12-flag_peaks.txt.gz
GSE118946_polII_peaks.txt.ga

GSM6210722_Pooled_Input.bw
GSM6210718_H9-shControl_H3K27Ac.bw
GSM6210718_H9-shControl_H3K27Ac_peaks.txt.gz
GSM6210719_H9-shZBTB12_H3K27Ac.bw
GSM6210719_H9-shZBTB12_H3K27Ac_peaks.txt.gz
GSM6210720_H9-shControl_HDAC1.bw
GSM6210720_H9-shControl_HDAC1_peaks.txt.gz
GSM6210721_H9-shZBTB12_HDAC1.bw
GSM6210721_H9-shZBTB12_HDAC1_peaks.txt.gz

Genome browser session
(e.g. UCSC)

##Reference genome is hg19version from UCSC database

Methodology

Replicates

no replicates for each sample, PolII was used as positive control and Input as the negative control

Sequencing depth

	Total # of reads	Uniquely mapped reads	Length of reads	Single- or Paired-end
ZBTB12_Flag	36,733,142	32,474,644	150	paired end
Input	38,360,480	34,735,710	150	paired end
PolII	34,719,481	31,311,931	150	paired end
H9-input	183,224,598	124,568,895	150	paired end
shControl_H3K27Ac	224,921,410	150,951,443	150	paired end
shZBTB12_H3K27Ac	171,148,100	112,964,889	150	paired end
shControl_HDAC1	203,129,254	123,035,205	150	paired end
shZBTB12_HADC1	220,000,244	131,611,961	150	paired end

Antibodies

-AntiPolII (for chromatin immunoprecipitation), Sigma-Aldrich, 05-623: The antibody guarantee covers the use of the antibody for ChIP-application. The antibody has been referenced in more than 40 publications. https://www.merckmillipore.com/product/Anti-RNA-polymerase-II-Antibody-clone-CTD4H8,MM_NF-05-623

-Anti-FLAG (for chromatin immunoprecipitation), Cell signaling Technologies, 14793: The antibody guarantee covers the use of the antibody for ChIP application. The antibody has been referenced in 208 publications. https://www.cellsignal.com/products/primary-antibodies/dykddddk-tag-d6w5b-rabbit-mab-binds-to-same-epitope-as-sigma-s-anti-flag-m2-antibody/14793

-Anti-HDAC, Active Motif, 40967: The antibody guarantee covers the use of the antibody for ChIP application. The antibody has been referenced in 5 publications. https://www.activemotif.com/publications

-Anti-H3K27ac, Active Motif, 39133: The antibody guarantee covers the use of the antibody for ChIP application. The antibody has been referenced in 80 publications. https://www.activemotif.com/publications

Peak calling parameters

For ZBTB12 and PolII (macs2 callpeak -t *.rmdup.bam -c Input.rmdup.bam -f BAM -g hs -n $prefix -B -q 0.01 --outdir $prefix)
For H3K27ac and HDAC1 (macs2 callpeak -t *.rmdup.bam -c Input.rmdup.bam -f BAM -g hs -n $prefix -B -q 0.01 --broad --outdir

Data quality

	total # peaks	q <0.01
ZBTB12_Flag	3,629	3,629
PolII	32,077	32,077
shControl_H3K27Ac	60,208	60,208
shZBTB12_H3K27Ac	74,680	74,680
shControl_HDAC1	43,309	43,309
shZBTB12_HADC1	45,743	45,743

Software

Bwa(version 0.7.13), MACS2, HOMER(version3.12)
